# Towards the detection of copy number variation from single sperm sequencing in cattle

**DOI:** 10.1186/s12864-022-08441-8

**Published:** 2022-03-17

**Authors:** Liu Yang, Yahui Gao, Adam Oswalt, Lingzhao Fang, Clarissa Boschiero, Mahesh Neupane, Charles G. Sattler, Cong-jun Li, Eyal Seroussi, Lingyang Xu, Lv Yang, Li Li, Hongping Zhang, Benjamin D. Rosen, Curtis P. Van Tassell, Yang Zhou, Li Ma, George E. Liu

**Affiliations:** 1 Animal Genomics and Improvement Laboratory, Henry A. Wallace Beltsville Agricultural Research Center, Agricultural Research Service, USDA, Beltsville, MD 20705 USA; 2 College of Animal Science and Technology, Sichuan Agricultural University, Chengdu, 611130 China; 3 Department of Animal and Avian Sciences, University of Maryland, College Park, MD 20742 USA; 4 Select Sires Inc, 11740 U.S. 42 North, Plain City, OH 43064 USA; 5 MRC Human Genetics Unit at the Institute of Genetics and Cancer, The University of Edinburgh, Edinburgh, EH4 2XU UK; 6 Agricultural Research Organization (ARO), Institute of Animal Science, HaMaccabim Road, P.O.B 15159, 7528809 Volcani CenterRishon LeTsiyon, Israel; 7 Innovation Team of Cattle Genetic Breeding, Institute of Animal Sciences, Chinese Academy of Agricultural Sciences, Beijing, 100193 China; 8 Key Laboratory of Agricultural Animal Genetics, Breeding and Reproduction of Ministry of Education & College of Animal Science and Technology, Huazhong Agricultural University, Wuhan, 430070 China

**Keywords:** Cattle, Single sperm sequencing, Copy number variation

## Abstract

**Background:**

Copy number variation (CNV) has been routinely studied using bulk-cell sequencing. However, CNV is not well studied on the single-cell level except for humans and a few model organisms.

**Results:**

We sequenced 143 single sperms of two Holstein bulls, from which we predicted CNV events using 14 single sperms with deep sequencing. We then compared the CNV results derived from single sperms with the bulk-cell sequencing of one bull’s family trio of diploid genomes. As a known CNV hotspot, segmental duplications were also predicted using the bovine ARS-UCD1.2 genome. Although the trio CNVs validated only some single sperm CNVs, they still showed a distal chromosomal distribution pattern and significant associations with segmental duplications and satellite repeats.

**Conclusion:**

Our preliminary results pointed out future research directions and highlighted the importance of uniform whole genome amplification, deep sequence coverage, and dedicated software pipelines for CNV detection using single cell sequencing data.

**Supplementary Information:**

The online version contains supplementary material available at 10.1186/s12864-022-08441-8.

## Background

Copy number variation (CNV) is defined as deletions, insertions, and duplications ranging from 50 base pairs (bp) to 5 million base pairs (Mbp) between any individuals [1]. CNV has been extensively studied in multiple species for its functional impacts on gene expression, such as altering gene dosage, disrupting coding sequence, or perturbing long-range gene regulation [2]. To date, CNV has been investigated in humans [1, 3–7], mice [8–10], and domesticated animals [11–20]. In cattle, we and others reported germline/inherited and somatic CNV using microarrays and short-read sequencing in breeds like Angus, Holstein, Hanwoo, Brown Swiss, Simmental, and Qinchuan [19, 21–28].

Recent breakthroughs in the development and application of single-cell sequencing technologies provide an avenue for dissecting population lineages and heterogeneity and understanding cell identity, differentiation, and function [29–34]. Single-cell DNA-seq (scDNA-seq) technologies produce data, which is ideal for detecting CNV or abnormal chromosome numbers (aneuploidy) on the single-cell level [35–37]. Because copy number aberrations (CNAs), which are pathogenic CNVs, play an important role in the initiation and progression of cancer, they have been intensively studied using single-cell sequencing in humans [38, 39]. Currently, multiple analysis tools are available for detecting CNVs in human scDNA-seq data, as reviewed recently [40].

However, no report has been published on the CNV identification on the single-cell level in livestock, including cattle. Here we sequenced and analyzed 143 single sperm genomes from two Holstein bulls, identifying thousands of candidate CNV events. We attempted to validate the single-sperm sequencing-based CNV results using the data derived from the diploid genome sequencing of one bull’s family trio. Since one mechanism of CNV formation is non-allelic homologous recombination (NAHR), a recent paper reported that NAHR leads to over two-thirds of the structural variation detected within the human genome [41]. We also investigated CNVs and their associated segmental duplications [2]. To the best of our knowledge, this is the first reported trial of single sperm genome sequencing in livestock, highlighting future CNV detection directions using scDNA-seq data and opening the door for studying individual sperm genome and male infertility.

## Results

### Sequencing of haploid sperms and diploid trio

#### Sequencing of sperms

We chose two bulls with different fertility capabilities (See Methods). Using the MALBAC method [42], we amplified and sequenced a total of 156 single sperm cells manually picked from two Holstein bulls’ semen. After quality control filtering, 143 sperm data (71 for Sample1 and 72 for Sample2) were kept for downstream analyses. The sequenced sperms had an average of 1.79 × genome coverage, and 16 of them were at ~ 4 × genome coverage, achieving an overall coverage of ~ 11.40% to ~ 41.35% of the genome, respectively (Table S1). On average, 98.18% of sequencing reads from single sperms were mapped on the bovine ARS-UCD1.2 genome.

#### Sequencing of the trio

For Samples1’s family trio diploid genomes, we sequenced bulk DNA samples extracted from ear punches of Sample1, its sire Sample1-sire, and dam Sample1-dam to approximately 40, 10, and 20 × genome coverage, respectively, with over 99% genome mapping rate and covering 96% genome sequence (Table S2).

### Segmental duplication analysis

**Fig. 1:**
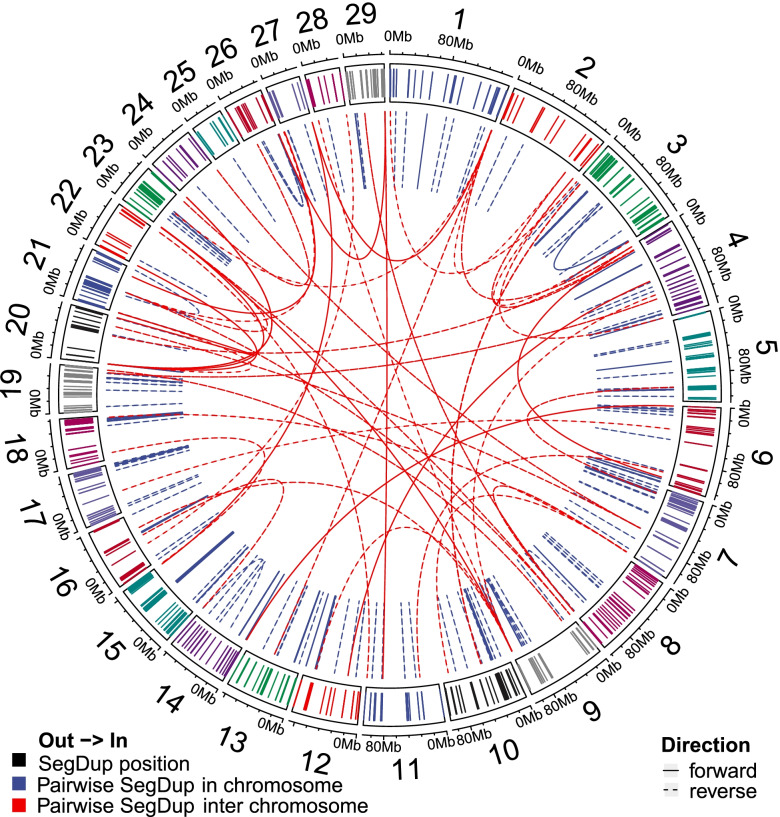
Landscapes of segmental duplications. A Circos plot generated by R (version 4.0.2) package circlize. From outside to inside: segmental duplication position, pairwise intrachromosomals, and interchromosomal SegDup events. Solid lines denote the pairwise SegDup with the same orientation, while dotted lines for reversed orientation. Only SegDups with lengths larger than 10 kb, sequence identity of more than 90% are shown

Delineation of the recent duplication events at the genomic-sequence level, particularly sequences located at their junctions [43], may provide insight into their mechanism of origin. Because SDquest can detect recent and ancient segmental duplication (SegDup) [44], we applied it to the latest bovine ARS-UCD1.2 genome assembly. A total of 27,560 pairwise SegDup sequence fragments were reported by SDquest, with 49,126 unique nonredundant fragment regions (Table S3). Among them, 12,400 (44.99%) and 3,374 (12.24%) pairwise SegDups have sequence identity larger than 80% and 90%, respectively. Also, 17,477 (63.41%) pairwise SegDup sequence fragments are reversed in their orientations on the chromosomes, while 16,621 (60.31%) are interchromosomally distributed (Fig. 1). After merging neighboring pairwise SegDup sequence fragments, we detected a total of 9,445 SegDup regions, covering 2.89% of the bovine genome (71,877,120 bp) (Table S4). As shown in Table S5, chr3 has the highest count of SegDup regions (600), chr5 has the largest length of SegDup regions (5,004,378 bp), and chr29 has the largest percentage of SegDup coverage (7.42%).

Following our previous study [45], we analyzed repetitive sequence contents in and near SegDup regions (Table S6, Methods). We evaluated the repeat content of duplicated sequence, 20 kb flanking sequence, and the whole genome. As reported before [35], SINE Alu repeats were associated with human segmental duplications, but we did not find SINE enrichment was enriched for bovine segmental duplications. However, we detected two clear patterns regarding repeat content. While LINE content remains similar, DNA and SINE repeat content of most duplications are reduced. We observed a reverse trend for LTR and satellite repeat sequences, even though the fold change for LTR is only 1.25 (Table S6, Random simulation test, *P-value* < 0.001). Bovine segmental duplications show a 2.84-fold enrichment for satellite repeat content and a 2.03-fold elongation for satellite repeat average length over the genome average (Table S6), agreeing with our earlier observation [45].

We also performed gene annotation for those SegDup regions and found 3,724 SegDups overlapping with 2,969 genes, which were significantly enriched (adjusted *P-value* < 0.05) in the GO term of GTPase activity and 12 KEGG pathways, such as metabolism of xenobiotics by cytochrome P450 and antigen processing and presentation (Table S7), again agreeing with our previous cattle results and the results from other species [8–10, 45]. When compared with the cattle QTL database [46], we found a total of 837 QTLs intersected with 425 SegDups. We also found that eight QTLs were significantly enriched (adjusted *P-value* < 0.05 after the Benjamini–Hochberg correction for multiple testing) for animal reproduction and health traits, such as conception rate, inseminations per conception, stillbirth, bovine respiratory disease susceptibility, and others (Table S7).

### Copy number variations in sperms and trio genomes

**Fig. 2:**
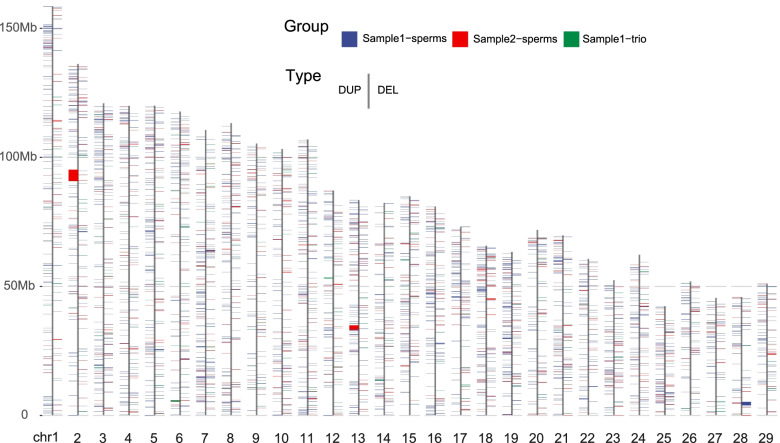
Landscapes of copy number variations. DUP and DEL events are shown on the left and right sides of a chromosome, respectively. Locations of copy number variations are shown for Sample1-sperms (blue), Sample2-sperms (red), and Sample1-trio (green)

**Fig. 3:**
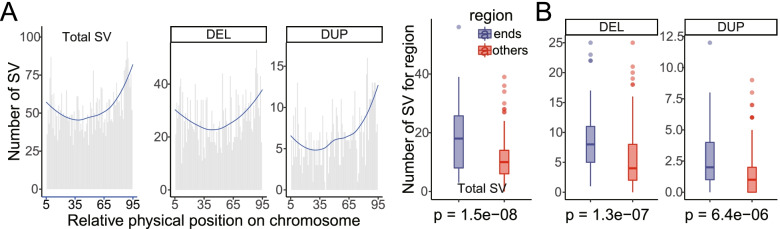
Landscapes of segmental duplications and copy number variations. **A** CNV count distributed from 5 to 95% of chromosomal arms. The Y-axis of the left panel represented the total structural variation count, including BND, DEL, DUP, and INV. The other two figures are for DEL only or DUP only, respectively. **B** Total structural variation, DEL, and DUP are significantly enriched in two ends of chromosomes. The number of structural variation, DEL, and DUP events in both ends of each chromosome (5 Mb after removing the terminal 5%) are significantly larger than of other chromosome regions. All *P*-values are calculated by the Wilcoxon test

Using single sperms with deep sequencing from Sample1 (*n* = 8) and Sample2 (*n* = 6), as well as Sample1 trio somatic samples, we detected a total of 5,646 CNVs (ranging from 50 bp to 5 Mb), including 1,307 break end (BND), 2,779 deletion (DEL), 877 duplication (DUP), and 683 inversion (INV) events (Table 1, Table S8, and Table S9). Totally 0.27% of autosomes were covered by 6.73 Mb length of CNV (Table S10). We then focused on CNVs (i.e., DEL and DUP), which are shown in Fig. 2 and Fig. S1. Similar to the recombination maps derived from the same sequence data (Yang et al., 2021 submitted), CNV distributions are significantly enriched in the two ends of chromosomes (Fig. 3). This result was also in line with the human results from two recent large-scale CNV discovery studies [47, 48].

**Table 1:** Statistics of copy number variation by group

ID	Count					Length (kb)				Genome covered			
	BND	DEL	DUP	INV	Total	DEL	DUP	INV	Total	DEL	DUP	INV	Total
Total	1307	2779	877	683	5646	9724.72	16,140.42	598.91	26,464.05	0.391%	0.648%	0.024%	1.063%
Total sperms	1262	2495	859	666	5282	9048.34	14,305.07	472.94	23,826.35	0.363%	0.575%	0.019%	0.957%
Sum sample1-sperms	919	1714	732	654	4019	6892.22	6476.01	378.86	13,747.09	0.277%	0.260%	0.015%	0.552%
Sum sample2-sperms	343	781	127	12	1263	2156.12	7829.06	94.08	10,079.26	0.087%	0.314%	0.004%	0.405%
Total sample1-trio	45	284	18	17	364	676.38	1835.34	125.98	2637.70	0.027%	0.074%	0.005%	0.106%

After removing the CNV singletons (i.e., DEL or DUP occurred only once in one sample), we obtained 433 DEL and DUP events in a total length of 1,736.6 kb. Within them, 4.16% (18 out of 433, 7.17% for length) of CNVs were detected in all three groups. Near 51.04% (221/433, 71.00% for length) were found in two groups. Among those, 10.39% SV (45/433, 15.97% for length) were shared in Sample1-sperms and Sample1-trio, and 36.49% of the total events (158/433, 32.22% for length) were shared in Sample1-sperms and Sample2-sperms (Fig. S2 and Table S10). CNVs unique to Sample1-trio accounted for only 3.46% events (15/433) (2.93% for length), while CNVs unique to Sample1-sperms accounted for 28.41% events (123/433) (16.16% for length) (Fig. S2 and Table S10). These results indicated that a larger number of CNVs detected in sperms did not overlap with the trio CNVs. Those 433 CNVs mapped 968 genes, which were significantly enriched (adjusted *P-value* < 0.05) with four GO terms of cell migration and one KEGG pathway of pancreatic secretion (Table S11).

In addition, we analyzed the repeat content in and near 2,485 nonredundant CNV regions, similar to what we did for SegDup as described above (Table S12). We tested two flanking window sizes: 5 and 20 kb. For Sample1 sperm, Sample2 sperm, and Sample1 diploid CNVs, we consistently observed that SegDup (3.67–13.79 folds) and satellites (up to 5.64 folds) were enriched in CNVs (all *P-values* < 0.001). Within the 5 kb flanking regions, the enrichment folds of SegDups and satellites fall to 1.74–2.91, 0.45–2.42 folds, respectively. They gradually decrease to the genome average as flanking windows around the CNV increase to 20 kb.

## Discussion

Single-cell sequencing and analysis are still facing multiple grand challenges [49]. To the best of our knowledge, this is the first trial of single sperm sequencing in the livestock, and we will discuss what was achieved and what needs further improvement.

It is generally accepted that the de novo CNV event is infrequent. By mapping each sperm’s sequencing data to the reference genome, our results focused on the germline/inherited CNVs, which are the common CNV events shared by single sperms. Using the CNV results derived from the trio bulk-cell sequencing as the ground truth, we estimated shared and unique percentages among the three groups (Sample1-sperms, Sample2-sperms, and Sample1-trio). To our disappointment, only 10.39% of CNVs detected in Sample1-sperms were shared with its family trio, while 36.49% were shared between Sample1-sperms and Sample2-sperms. Thus, it is possible that CNVs only called in single cells were less reliable. We suspect the following systematic factors may contribute to these discrepancies: 1) uneven whole genome amplification, 2) low sequence coverage, and 3) suboptimal pipelines and their parameters.

As expected, scDNAseq is limited by its DNA amount: a single sperm contains 3 pg of DNA, not enough for whole-genome sequencing. Therefore, scDNAseq template amplification and library preparation are needed. As shown previously [50], these steps could severely impact the performance of CNV detection when whole genome amplification is uneven and/or sequence coverage is low. Additionally, the bioinformatics pipelines also influenced the performance of CNV detection. Ideally, read depth should be a better strategy given the low sequencing coverage, as compared to the pair-end and split-read approaches. As reviewed before [40], to correct for the first two factors, existing scDNAseq CNV read depth detection pipelines need to divide the genome into bins or windows first. They will then perform GC correction and mappability correction to obtain normalized reads depths (Figs. S3 and S4). Finally, they will need to remove outlier bins and outlier cells. The outlier bins often have an unusually high read count and occur near the centromere and telomere of each chromosome. The outlier cells often are low in signal-to-noise ratio or low in sequence coverage.

However, most of the existing pipelines are designed for the human genome [40], and it will take a great effort to fully customize and optimize them for livestock like cattle. In this study, CNVs were called using LUMPY [51], which was not designed for scDNA-seq data. It is also better to simultaneously apply a method to multiple samples to call germline/inherited CNVs to achieve better sensitivity and accuracy as the recently published method CHISEL did for human data [52]. Then CNV genotyping could then be performed on individual sperm cells. Our pipeline processed each sample separately using an integrated algorithm combining pair-end, split-read, and read depth. It did not specifically remove the outlier bins or the outlier cells, as no such data exists for cattle. However, our rationale for using LUMPY was that although we had a low average coverage and a low read depth for individual sperm genomes, we sampled the same genomes multiple times, through different sperms, with a total accumulating read depth of 56.99 × and 43.68 × . Therefore, merging reads across diffident sperms, i.e., pseudo bulk sequencing, should yield relatively confident results. In the future, we plan to adopt existing human pipelines to alleviate the impacts of these systematic factors on CNV calling in cattle.

During meiosis, chromosome missegregation can cause aneuploidy. Using Sperm-seq, Bell et al. sequenced 31,228 human sperm genomes from 20 men, identifying crossovers and other genomic anomalies [37]. They discovered that human sperm donors had aneuploidy rates ranging from 0.01 to 0.05 aneuploidies per gamete [37]. Due to the limited sample size and probably the signal-to-noise ratio, no aneuploidy was detected in this study.

Finally, Ebert et al. recently reported that over two-thirds of CNV detected within the human genome were associated with NAHR, mediated by repetitive sequences, such as segmental duplications and common repeat elements [41]. It was encouraging that our cattle segmental duplication and CNV flanking sequence analysis results also showed they are significantly enriched for each other and satellite repeats, despite the suboptimal data quality due to the abovementioned factors. In summary, we sequenced single sperms in cattle, performed an initial CNV detection, and found a distal chromosomal distribution pattern, which agreed with previous results derived from cattle bulk-cell sequencing or human studies. In the meantime, our results also highlighted the importance of the uniform whole genome amplification, deep sequence coverage, and dedicated software pipelines for CNV detection using scDNA-seq data.

## Methods

### Sample collection and whole genome amplification and sequencing

We chose two Holstein bulls with different fertility capabilities: Sample1 has a DPR (daughter pregnancy rate) PTA value of 0.0, reliability of 0.99, estimated from 6,528 daughters. In contrast, Sample2 has a DPR PTA value of -3.2, reliability of 0.99, estimated from 15,314 daughters. Somatic tissue (ear punch) samples of Holstein Sample1, together with its parent somatic tissues, were donated by Select Sires, Inc (Plain City, OH, USA). Semen samples were freshly collected by Select Sires, Inc. in its routine artificial insemination semen straw production. After receiving them under liquid nitrogen in USDA-ARS Animal Genomics and Improvement Laboratory (AGIL), we manually isolated a total of 156 sperm cells from two Holstein bulls (Sample1 with 73 sperm cells and Sample2 with 83 sperm cells). Briefly, isolated sperms were thawed in 37 ℃ water for 30-45 s and treated with 0.25% Trypsin–EDTA, followed by dilution with PBS + 1% BSA and washing twice. The sperms were further diluted to a proper resolution using PBS + 1% BSA on a petri-dish, and active single sperms were picked up manually by pipetting into a reaction tube under a micromanipulator as described previously [42]. Whole-genome amplification was performed on single cells according to the manufacturer’s protocol, using the Single Cell Whole Genome Amplification Kit (Yikon Genomics, Shanghai, China) developed from the Multiple Annealing and Looping Based Amplification Cycles (MALBAC) method [35]. In brief, a single sperm was initially analyzed and pre-amplified by primers supplied in the kit with 8 cycles with multiple annealing steps. PCR generated fragments with variable length at random starting positions for Illumina short-read sequencing. To evaluate the agreement rate of individual recombination from sperms and parents, we also sequenced the somatic diploid genomes of the trio, including Sample1 (Sample1-diploid) and its parents (Sample1-sire and Sample1-dam). Using their somatic ear punch tissues, we isolated their diploid genomes using a QIAGEN DNA extraction kit. DNA samples extracted from the donor and his parents' ear skin samples were then used to prepare sequencing libraries using standard Illumina protocol and sequenced on an Illumina HiSeq 2000/NextSeq 500 sequencing platform.

### Identification of segmental duplications and enrichment test

We utilized software SDquest v0.1 [44] for detecting segmental duplications (SegDup, also known as low copy repeats) and constructing the breakpoint graph of these mosaic SegDups, based on the repeat masked ARS-UCD1.2 reference downloaded from ENSEMBL (ftp://ftp.ensembl.org/pub/release-102/fasta/bos_taurus/dna/). We compared the repeat content of SegDups, CNVs (DEL or DUP), or 5 kb, 20 kb flanking regions (5kbF, 20kbF). For CNVs, we combined the SegDups and repeats from UCSC Table Browser (https://genome.ucsc.edu/cgi-bin/hgTables). Length, Count, Average Length, Length%, and Count/Mb of repeat content for SegDups, CNVs, 5kbF, or 20kbF were based on these repeat overlapped with regions, Length% denotes the proportion of repeat length overlapped with SegDups/CNVs/5kbFRoCNVs in total SegDups/CNVs/5kbF/20kbF length, Count/Mb denotes the count of repeats overlapped with SegDups/CNVs/5kbF/20kbF divided by total SegDups/CNVs/5kbF/20kbF Mb. For enrichment, ratios were defined as Average Length, Length%, and Count/Mb of repeats in SegDups/CNVs/5kbF/20kbF divided by repeats in the genome. We determined the significance of the enrichment by 1,000 times simulating the SegDups/CNVs/5kbF/20kbF in random genome position with the same average and standard deviation length, which generated by function createRandomRegions from R v4.0.2 package regioneR. *P*-value refers to the frequency of simulated value larger than observed value divided by simulation times. The threshold was set as 0.05.

### Structural variation detecting

We employed LUMPY v0.2.13 [51], which integrated read-depth, read-pair, and split-read strategies, to detect structural variations in high coverage sperms. As recommended, LUMPY was internally implemented in a pipeline smoove (https://github.com/brentp/smoove) with shorter run-time and lower false-positive rate. Smoove was used to collect the best practices of LUMPY, such as generating empirical insert size statistics on each library in the BAM file, estimating the mean and standard deviation (SD) of the input parameters for LUMPY. From LUMPY, the four types of structural variations, including deletion (DEL), duplication (DUP), inversion (INV), and break end (BND), were reported for each sample. Due to the limitation for INV and BND detection, we focused on CNV (DEL plus DUP) in most of the analysis, after filtering away DEL and DUP with a length more than 5 Mb or short than 50 bp. For haploid sperms and diploid trio, we applied the following thresholds to filter out low-quality CNVs: the threshold of supporting read count for either paired-end event or split-read event must be more than 3/4 of the genome coverage, while the read count for the other type of split-read event must be more than 1 or paired-end event must be more than 3.

### Gene annotation and enrichment analysis

We mapped regions of interest to the bovine reference gene annotation of the ARS-UCD1.2 genome from ENSEMBL using BEDtools v2.26.0 [53]. The gene features included transcripts, exons, CDS, 3’-UTR, 5’-UTR, start codon, and stop codon. The Kyoto Encyclopedia of Genes and Genomes (KEGG) pathway and Gene Ontology (GO) enrichment were performed using the R (version 4.0.2) packages org.Bt.eg.db and clusterProfiler. We performed the quantitative trait loci (QTL) enrichment analysis using the Fisher exact test at animalgenome.org [46]. All enrichment *P-*values were also adjusted for multiple comparisons by Benjamini and Hochberg's (BH) algorithm.

## Supplementary Information

**Additional file 1: **

**Additional file 2: **

## Data Availability

The data that support the results of this research are available within the article and its Supplementary Information files. All other sequence data can be tracked in supplemental files. The single sperm sequencing data and the trio whole genome sequencing data were submitted to GEO under the accession number PRJNA691741 (https://dataview.ncbi.nlm.nih.gov/object/PRJNA691741?reviewer=kj8n0f06eekt1uck7726jijms3).

## Abbreviations

AGIL: Animal Genomics and Improvement Laboratory
CNV: Copy number variation
DPR: Daughter pregnancy rate
GWAS: Genome-wide association study
HMM: Hidden Markov model
INDEL: Short insertion and deletion
kb: Kilobase pairs
LD: Linkage disequilibrium
MALBAC: Multiple annealing and looping based amplification cycles
Mb: Megabase pairs
NAHR: Non-allelic homologous recombination
PCR: Polymerase chain reaction
PRDM9: PR domain-containing 9
QC: Quality control
QTL: Quantitative trait loci
SD: Standard deviation
SE: Standard error
SegDup: Segmental duplication
SNP: Single nucleotide polymorphism
