# Supplementary material for: Towards the detection of copy number variation from single sperm sequencing in cattle
Source: BMC Genomics. 2022 Mar 17;23:215. doi: 10.1186/s12864-022-08441-8 (PMC8928590; doi:10.1186/s12864-022-08441-8)
Supplement: Supplementary file 1 — Additional file 1: [file 12864_2022_8441_MOESM1_ESM.pdf]

Figure S1. The density of CNV in each chromosome.

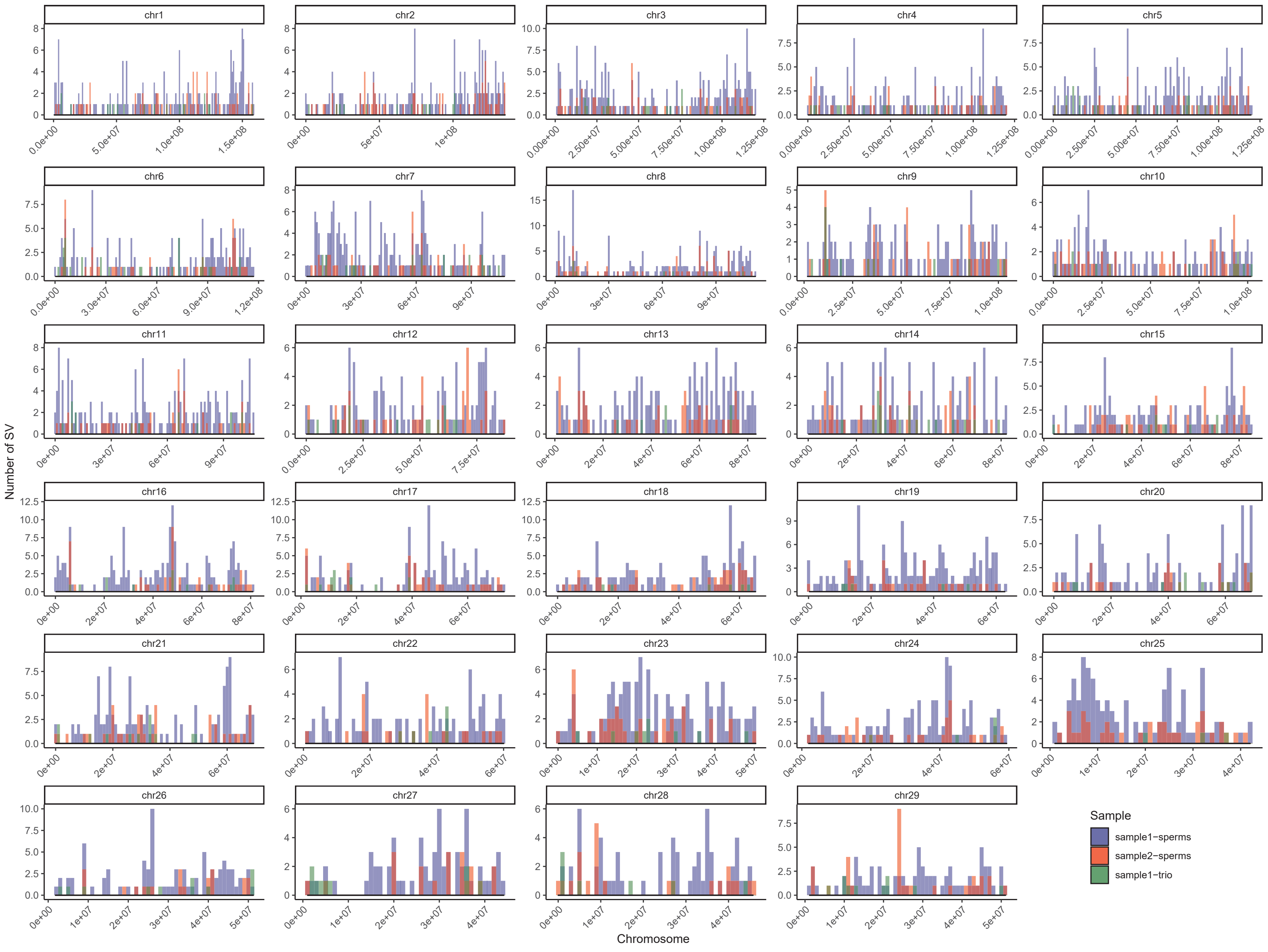

Figure S2. Venn plots for CNV (DEL and DUP) counts and lengths shared by trios and sperms.

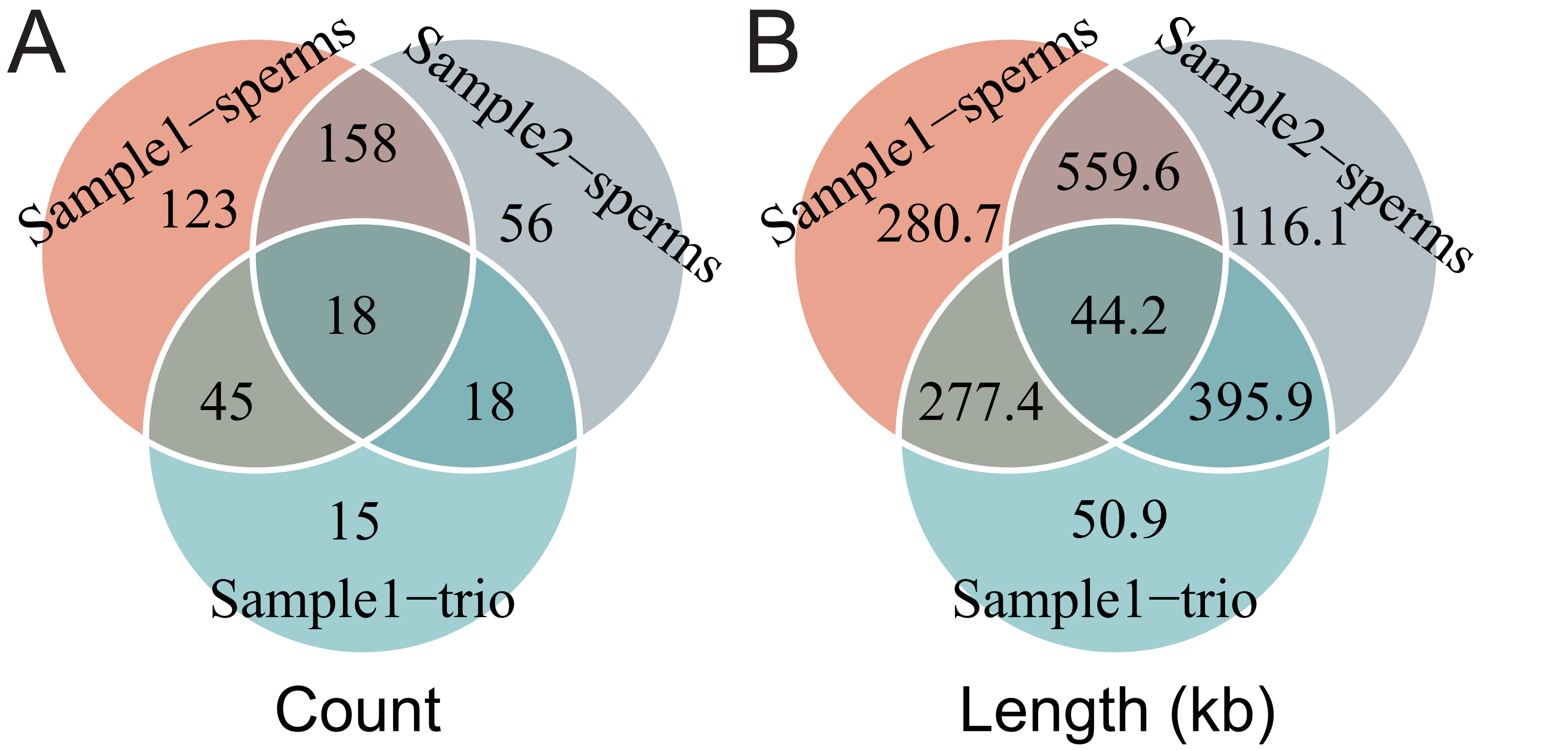

Figure S3. Normalized read depths along the genome in Sample1 trio.

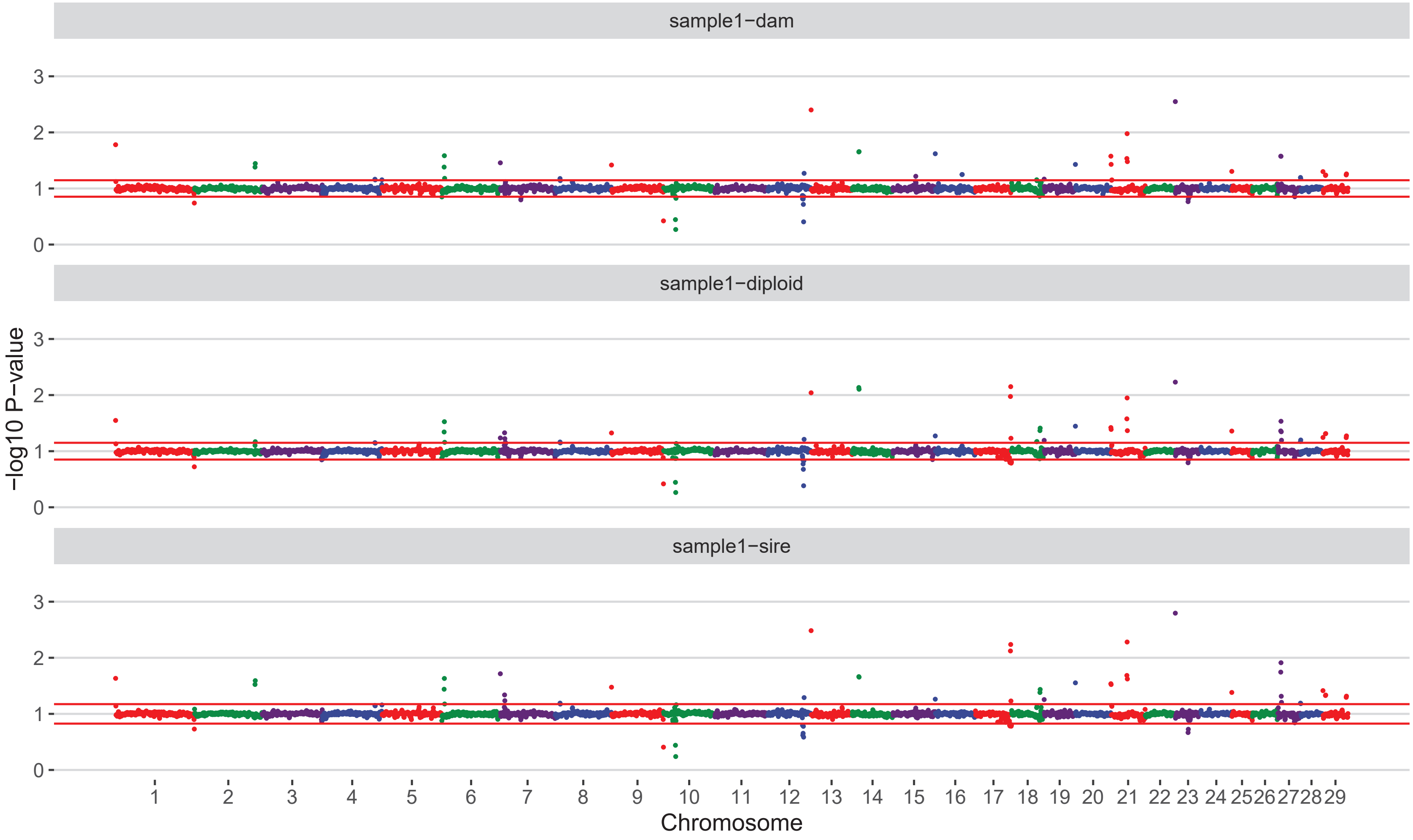

Figure S4. Normalized read depths along the genome in each sperm.

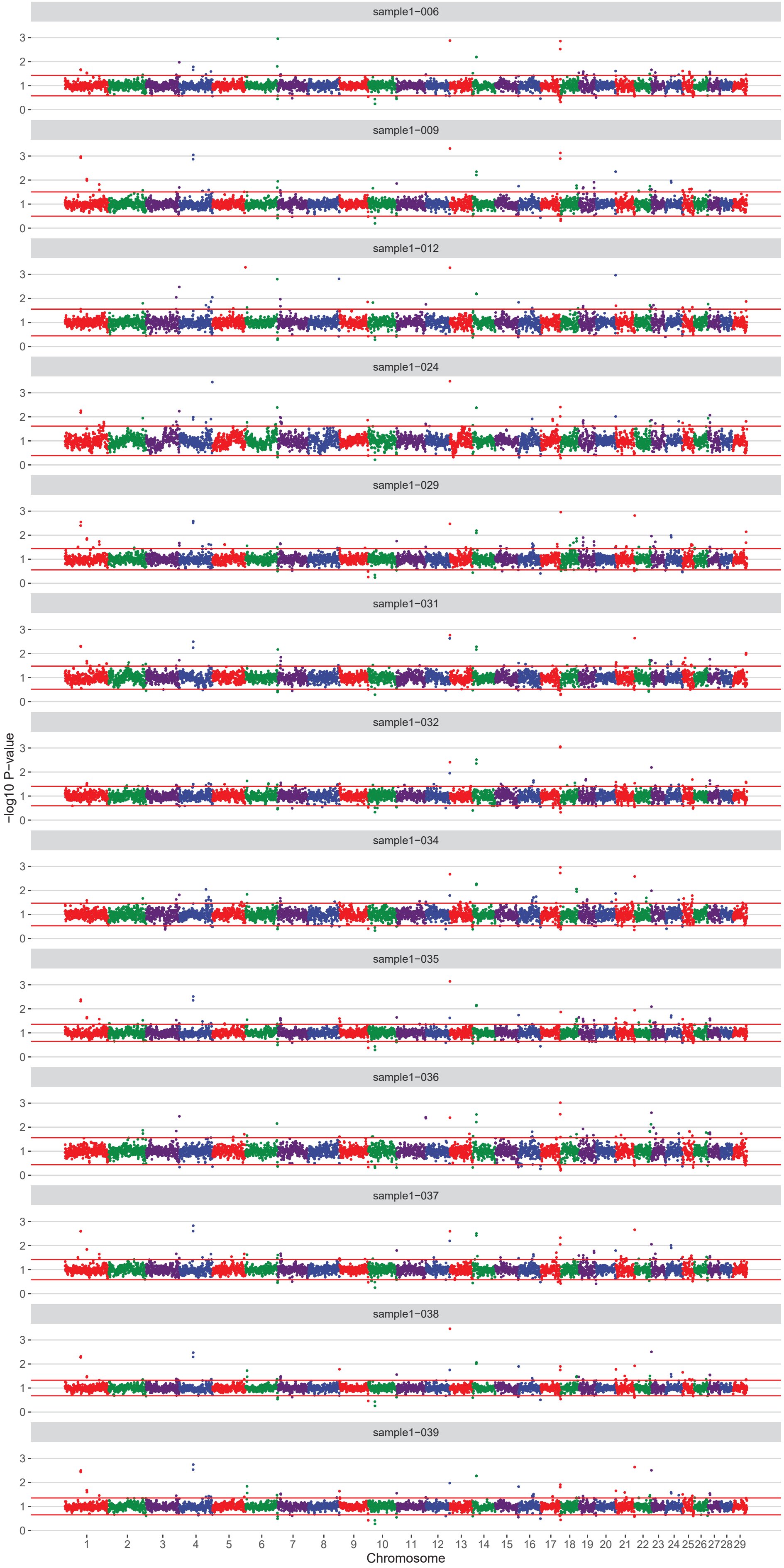

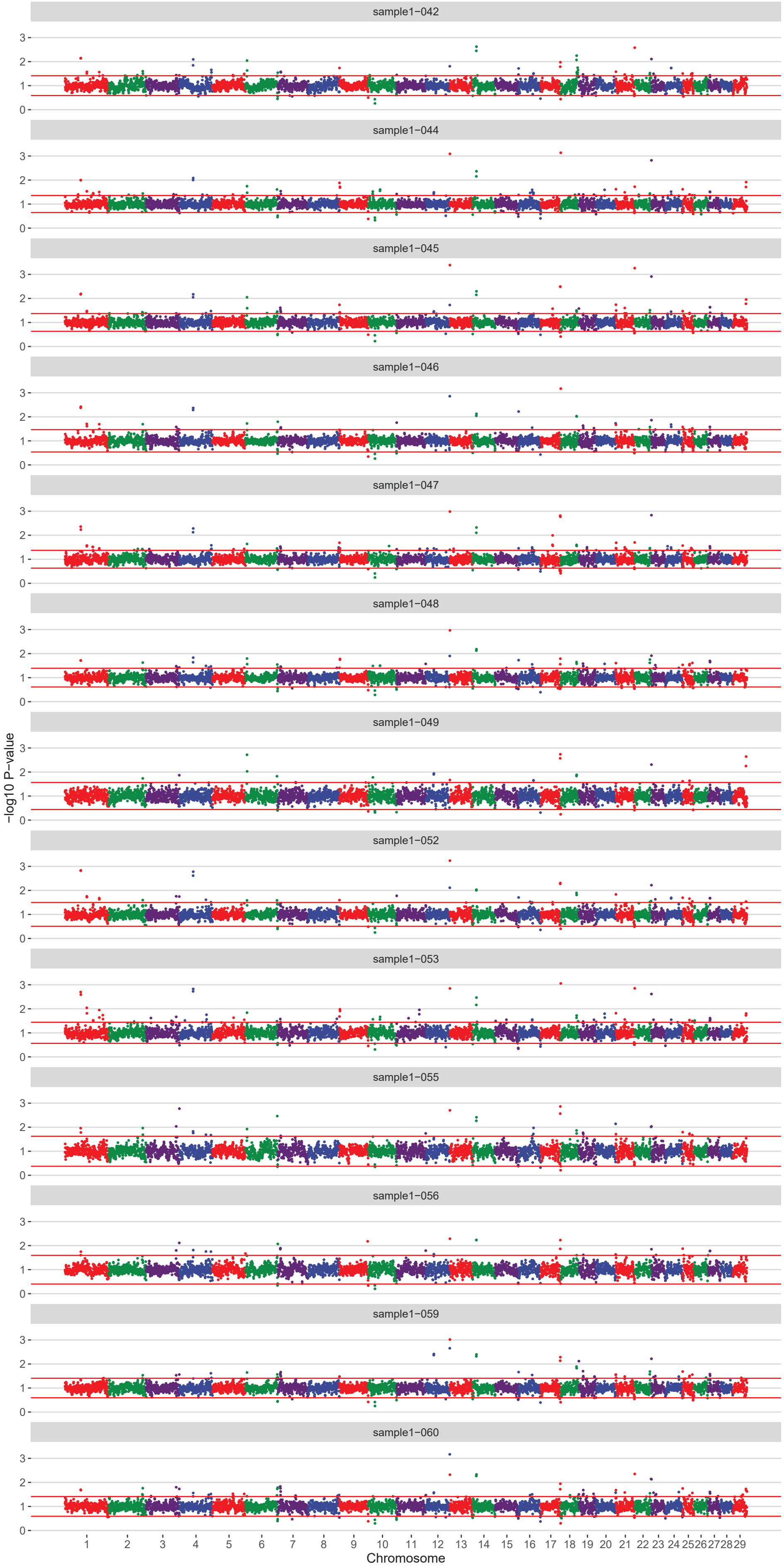
sample1−042
sample1−044
sample1−045
sample1−046
sample1−047
sample1−048
sample1−049
sample1−052
sample1−053
sample1−055
sample1−056
sample1−059
sample1−060
−log10 P−value
Chromosome
0
1
2
3
1
2
3
4
5
6
7
8
9
10
11
12
13
14
15
16
17
18
19
20
21
22
23
24
25
26
27
28
29

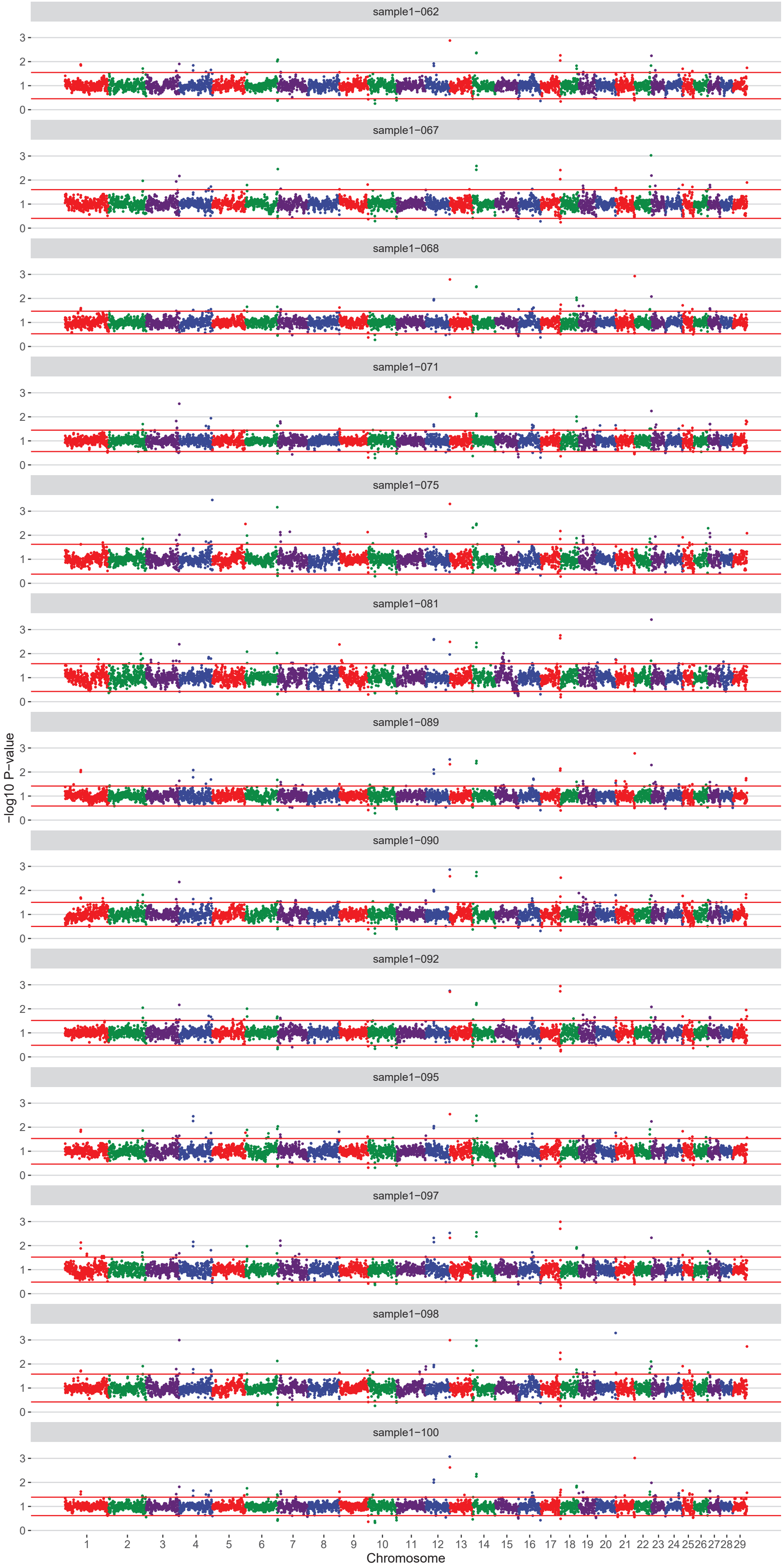

sample1−062
sample1−067
sample1−068
sample1−071
sample1−075
sample1−081
sample1−089
sample1−090
sample1−092
sample1−095
sample1−097
sample1−098
sample1−100
−log10 P−value
0
1
2
3
1 2 3 4 5 6 7 8 9 10 11 12 13 14 15 16 17 18 19 20 21 22 23 24 25 26 27 28 29
Chromosome

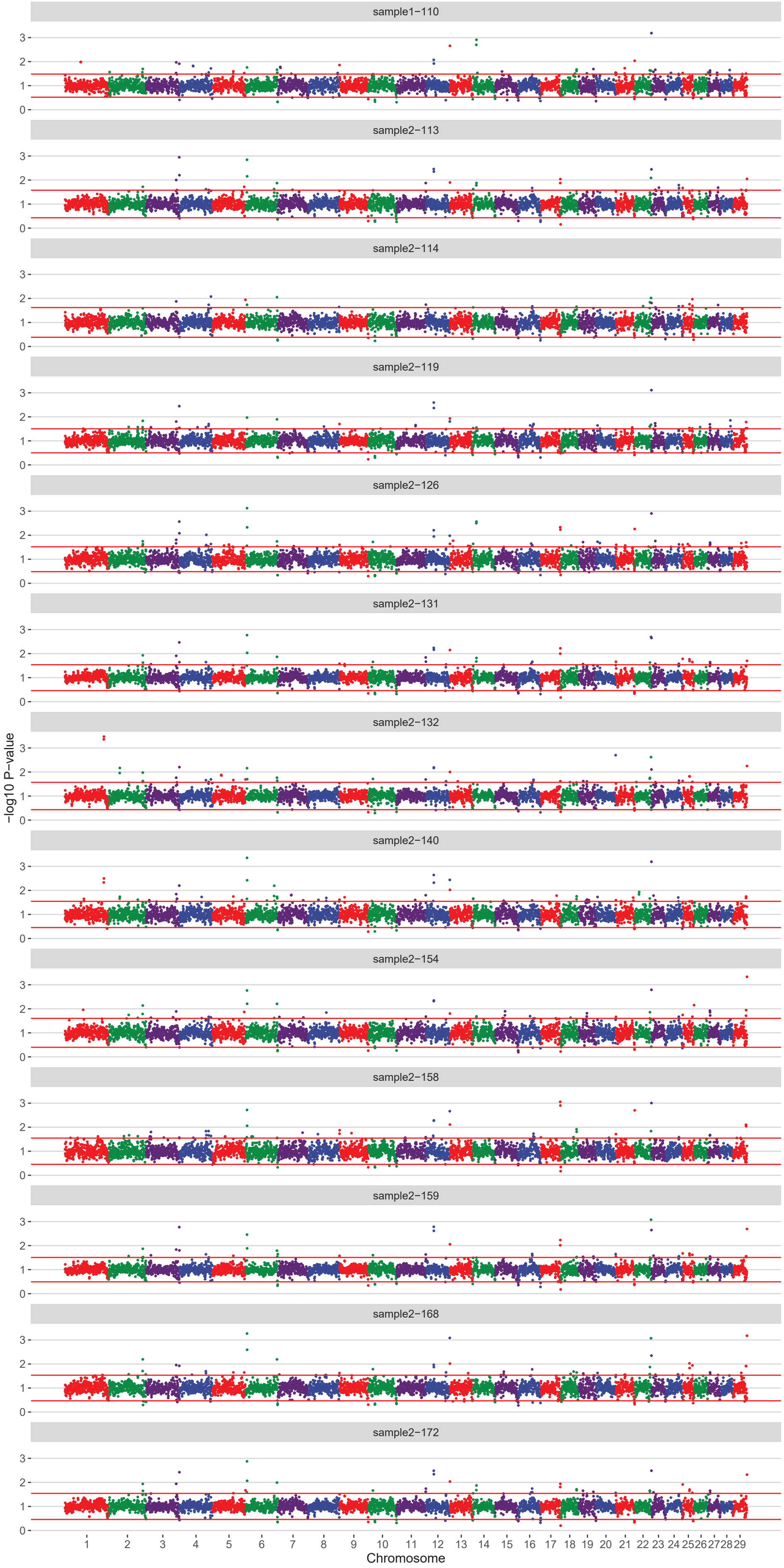

sample1−110
sample2−113
sample2−114
sample2−119
sample2−126
sample2−131
sample2−132
sample2−140
sample2−154
sample2−158
sample2−159
sample2−168
sample2−172
−log10 P−value
Chromosome
1 2 3 4 5 6 7 8 9 10 11 12 13 14 15 16 17 18 19 20 21 22 23 24 25 26 27 28 29

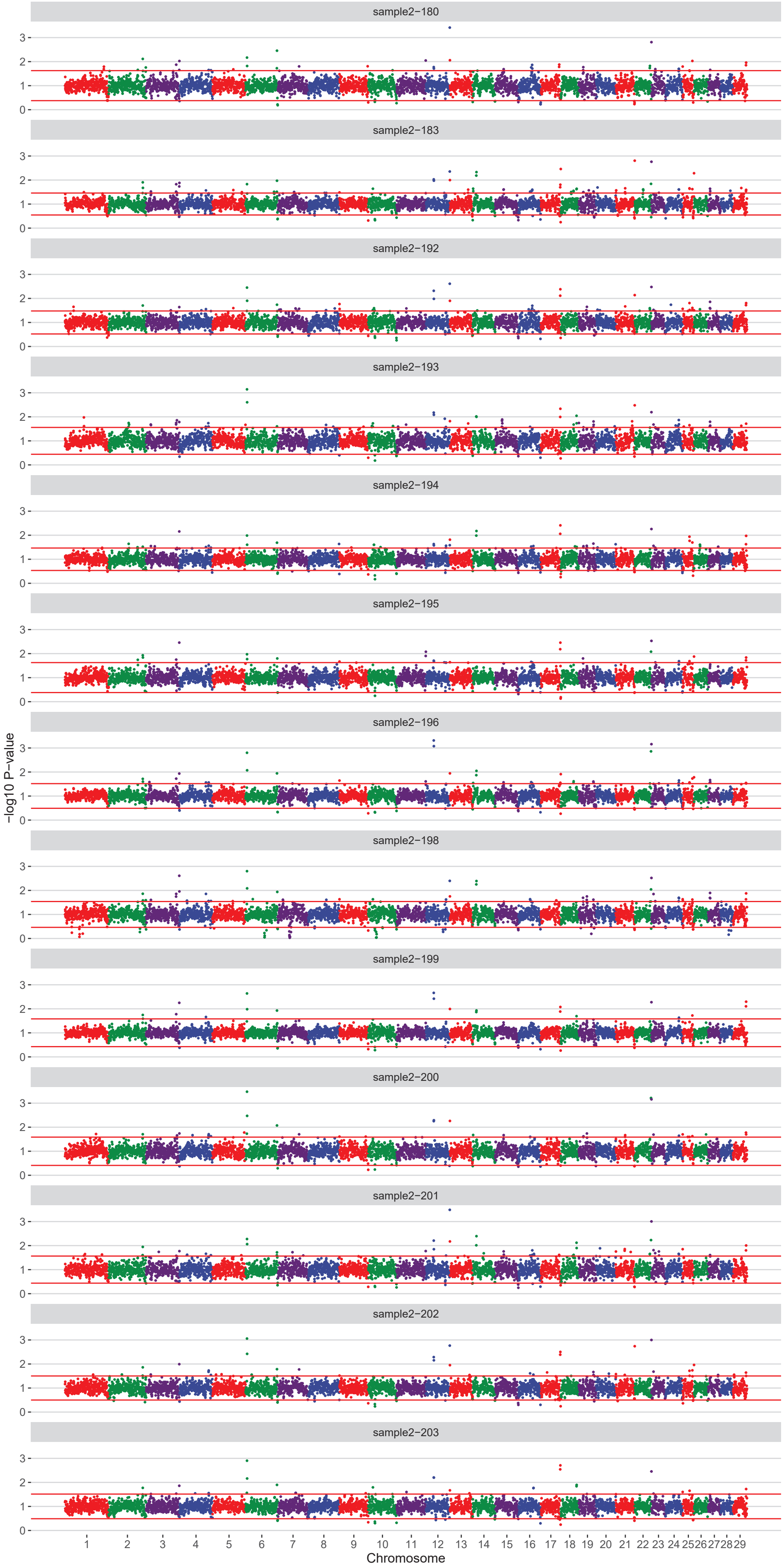
sample2−180
sample2−183
sample2−192
sample2−193
sample2−194
sample2−195
sample2−196
sample2−198
sample2−199
sample2−200
sample2−201
sample2−202
sample2−203
−log10 P−value
Chromosome
1 2 3 4 5 6 7 8 9 10 11 12 13 14 15 16 17 18 19 20 21 22 23 24 25 26 27 28 29

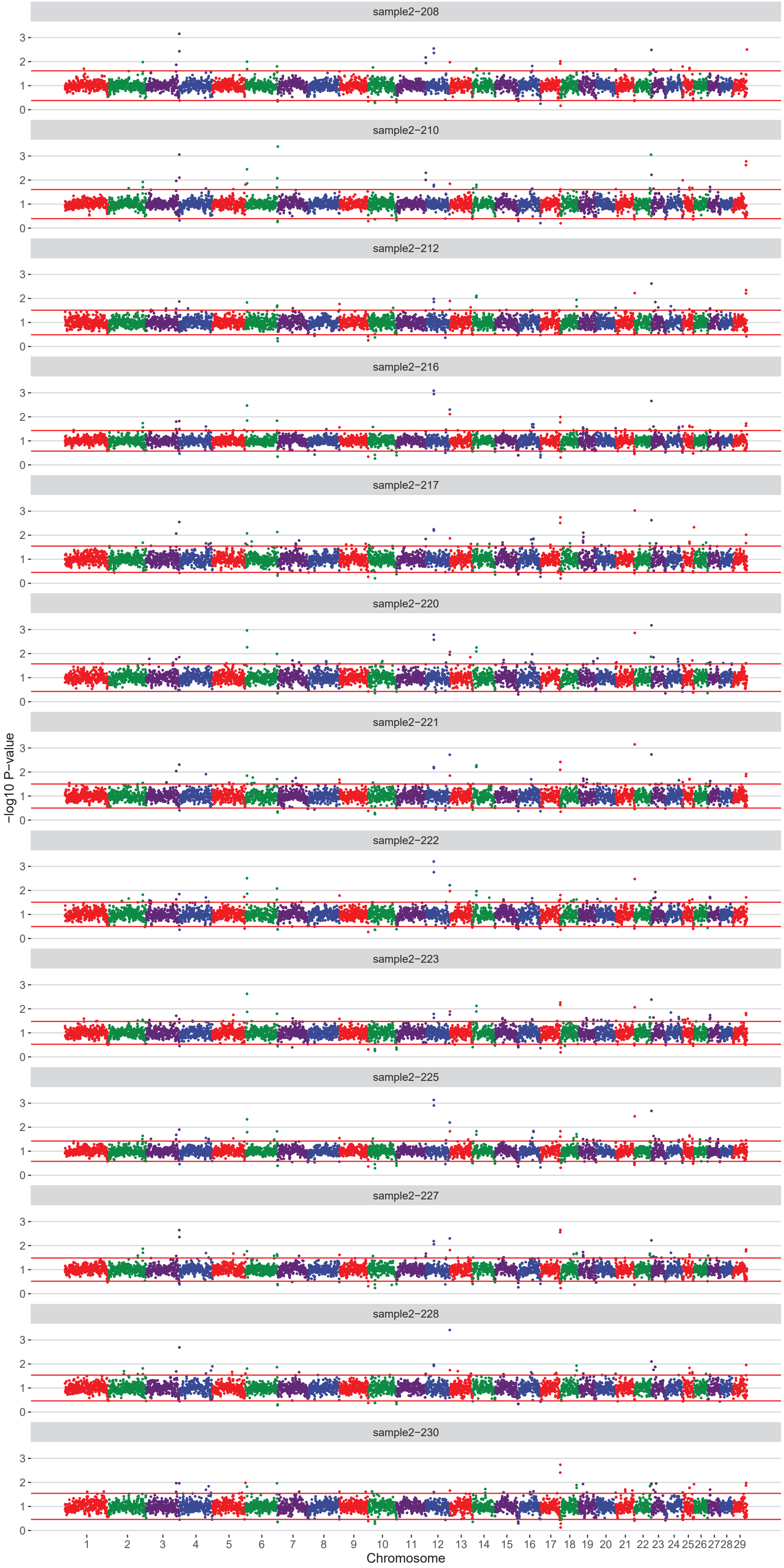
sample2−208
sample2−210
sample2−212
sample2−216
sample2−217
sample2−220
sample2−221
sample2−222
sample2−223
sample2−225
sample2−227
sample2−228
sample2−230
−log10 P−value
Chromosome
1 2 3 4 5 6 7 8 9 10 11 12 13 14 15 16 17 18 19 20 21 22 23 24 25 26 27 28 29
